# Cost-effectiveness of gefitinib, icotinib, and pemetrexed-based chemotherapy as first-line treatments for advanced non-small cell lung cancer in China

**DOI:** 10.18632/oncotarget.14310

**Published:** 2016-12-27

**Authors:** Shun Lu, Ming Ye, Lieming Ding, Fenlai Tan, Jie Fu, Bin Wu

**Affiliations:** ^1^ Department of Medical Oncology, Shanghai Chest Hospital, Shanghai Jiaotong University, Shanghai, China; ^2^ Department of Radiotherapy, Renji Hospital, School of Medicine, Shanghai Jiaotong University, Shanghai, China; ^3^ Betta Pharmaceuticals Co., Ltd., Hangzhou, China; ^4^ Medical Decision and Economic Group, Department of Pharmacy, Renji Hospital, School of Medicine, Shanghai Jiaotong University, Shanghai, China

**Keywords:** gefitinib, icotinib, EGFR mutation, cost-effectiveness, non-small cell lung cancer

## Abstract

Tyrosine kinase inhibitors of the epidermal growth factor receptor (EGFR) are becoming the standard treatment option for patients with advanced non-small cell lung cancer (NSCLC) harboring an EGFR mutation, but the economic impact of this practice is unclear, especially in a health resource-limited setting. A decision-analytic model was developed to simulate 21-day patient transitions in a 10-year time horizon. The health and economic outcomes of four first-line strategies (pemetrexed plus cisplatin [PC] alone, PC followed by maintenance with pemetrexed, or initial treatment with gefitinib or icotinib) among patients harboring EGFR mutations were estimated and assessed via indirect comparisons. Costs in the Chinese setting were estimated. The primary outcome was the incremental cost-effectiveness ratio (ICER). Sensitivity analyses were performed. The icotinib strategy resulted in greater health benefits than the other three strategies in NSCLC patients harboring EGFR mutations. Relative to PC alone, PC followed by pemetrexed maintenance, gefitinib and icotinib resulted in ICERs of $104,657, $28,485 and $19,809 per quality-adjusted life-year gained, respectively. The cost of pemetrexed, the EGFR mutation prevalence and the utility of progression-free survival were factors that had a considerable impact on the model outcomes. When the icotinib Patient Assistance Program was available, the economic outcome of icotinib was more favorable. These results indicate that gene-guided therapy with icotinib might be a more cost-effective treatment option than traditional chemotherapy.

## INTRODUCTION

Lung cancer is the most frequent malignancy and the most common cause of cancer-related death among males and females in the worldwide [1]. Based on the Chinese epidemiological data, the incidence (48.32 per 100,000) and mortality rate (39.27 per 100,000) of lung cancer in China were also high [2]. The disability-adjusted life years caused by lung cancer were documented in 2013 compared with 1990, especially among males [3]. Non-small cell lung cancer (NSCLC) manifests nearly 85% of all lung cancer cases [4], and about 46% of NSCLC cases are diagnosed as advanced disease at the time of presention [5]. Platinum-based chemotherapy have been recommended for patients with advanced NSCLC [6, 7]. However, clinical outcomes of chemotherapy is still poor, which showed the median overall survival (OS) time is approximately 10 months.

Small-molecule tyrosine kinase inhibitors (TKIs), such as erlotinib, gefitinib and afatinib, could specifically inhibit epidermal growth factor receptor (EGFR)-dependent pathway activity, could prolong OS and/or progression-free survival (PFS) in patients with advanced NSCLC and harboring an EGFR mutation [8, 9]. Thus, these EGFR-specific TKIs have been recommended in guidelines since 2010 for patients with newly diagnosed advanced NSCLC and harboring an EGFR mutation [6, 10]. Recently, icotinib, a novel EGFR-specific TKI originally developed in China, has been licensed for managing the Chinese newly diagnosed advanced NSCLC based on two large phase III randomized controlled trials (RCTs) [11, 12]. Owing to the lower cost, superior toxicity profile and equivalent efficacy of icotinib in comparison with erlotinib and gefitinib, icotinib appears to be a better alternative for Chinese patients with advanced NSCLC [12, 13]. However, utilizing both EGFR-specific TKIs may add a financial burden, especially when considering the cost of genetic screening in addition to the cost of the agents. Hence, it is necessary for health decision-makers, patients and physicians to determine the relative value of these potential first-line therapies. This study seeks to compare the economic outcomes of gene-guided first-line icotinib and gefitinib treatment with those of pemetrexed-containing chemotherapy for advanced NSCLC in the Chinese health care setting.

## RESULTS

### Base-case analysis

Compared to the control strategy (Table 1), the pemetrexed maintenance strategy, the gefitinib strategy, and the icotinib strategy yielded marginal QALYs of 0.094, 0.003 and 0.023, respectively. As the baseline comparator, the control strategy was the least expensive strategy. The pemetrexed maintenance, gefitinib, and icotinib strategies added costs of $9,519, $2,010, and $1,862, respectively, relative to the control strategy, resulting in ICERs of $104,657, $28,485, and $19,809 per QALY gained, respectively. When the PAP for icotinib and gefitinib became available, the ICERs for the icotinib and gefitinib strategies became to $15,451 and $22,577 per QALY gained, respectively.

**Table 1:** Summary of Cost ($) and Outcome Results in base-case analysis

Strategy	Cost	Progression-free LYs	Overall LYs	QALYs	Incremental cost per QALY*	Comments
PC (control strategy)	22,127	0.206	1.058	0.513	NA	
Pemetrexed maintenance strategy	31,646	0.300	1.208	0.604	104,657	Dominated
Gefitinib strategy	24,137	0.279	1.165	0.584	28,485	Dominated
Icotinib strategy	23,989	0.303	1.202	0.607	19,809	Dominance
Gefitinib strategy with PAP	23,721	0.279	1.165	0.584	22,577	Dominated
Icotinib strategy with PAP	23,580	0.303	1.202	0.607	15,451	Dominance

* Comparing with Control strategy

### Uncertainty analyses

**Figure 1:**
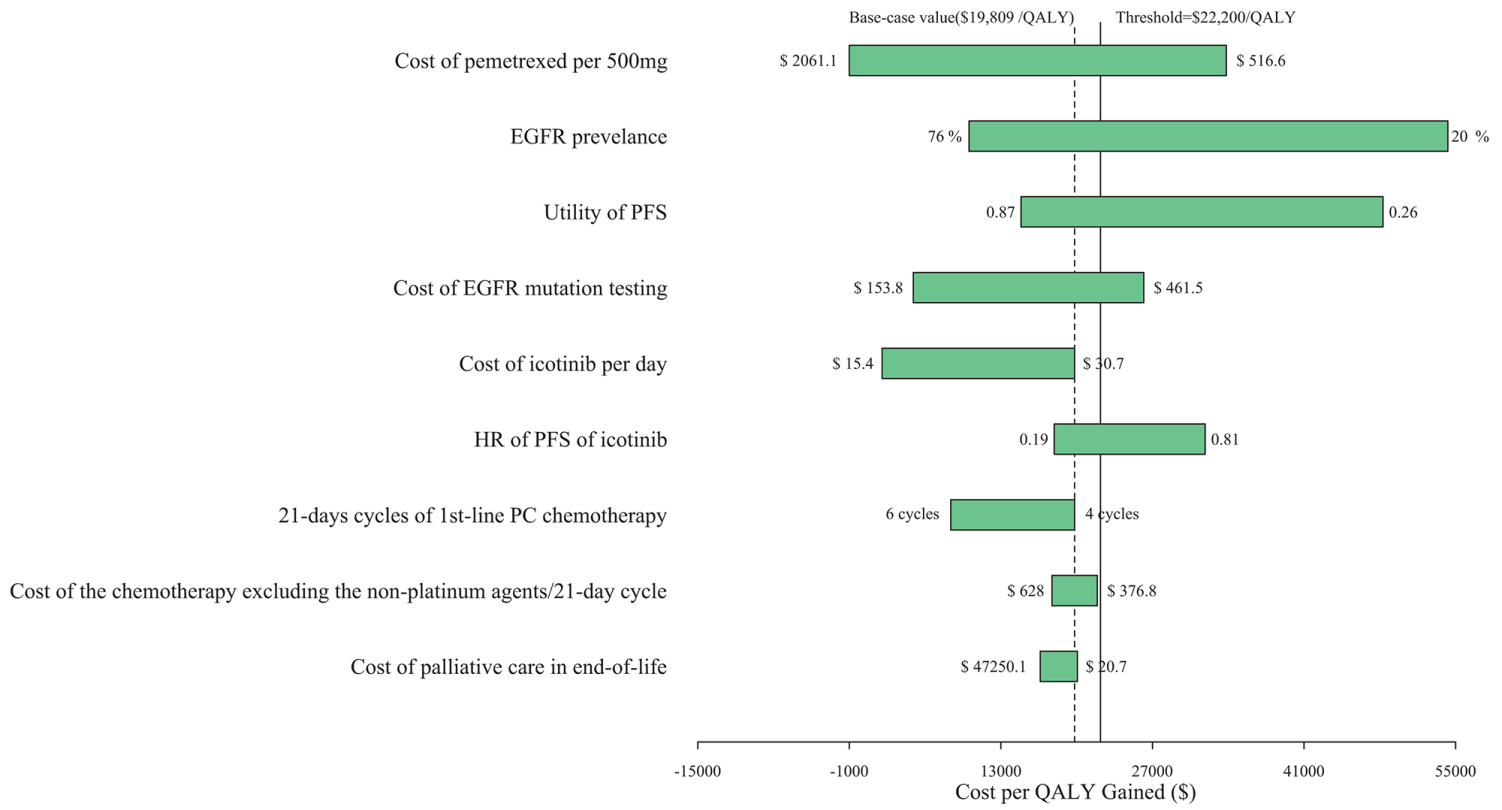
One-way sensitivity analyses for EGFR testing in combination with icotinib treatment in comparison with PC chemotherapy PC: pemetrexed plus cisplatin.

In the comparison between the icotinib and control strategies, the most influential variables were the cost of pemetrexed, the EGFR prevalence and the utility of PFS. Altering these parameters might yield substantial changes in the ICER (Figure 1). Other parameters, such as the cost of EGFR mutation screening and the HR of PFS of the icotinib versus the control strategy had a moderate or mild impact on economic outcomes.

**Figure 2:**
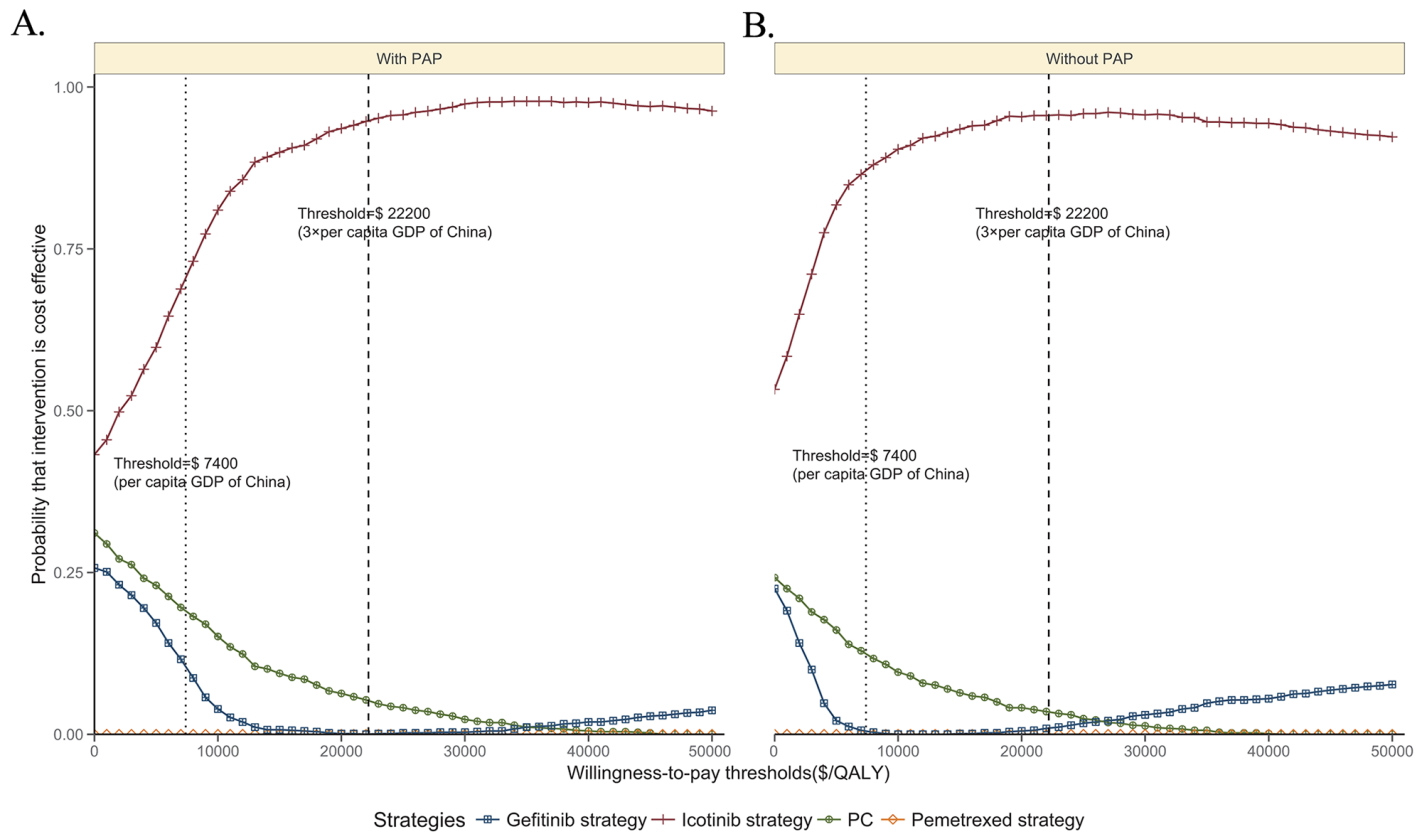
Acceptability curves comparing the cost-effectiveness of four competing strategies with the PAP **A**. or without the PAP **B**. QALY: quality-adjusted life-year. PC: pemetrexed plus cisplatin.

The CEACs are showed in Figure 2. Regardless of the availability of the PAP, the icotinib strategy showed cost-effectiveness in approximately 90% of the simulations, considering a cost-effectiveness threshold of $22,200 (3× the Chinese per capita GDP in 2015).

## DISCUSSION

This study to firstly examine the cost-effectiveness of four licensed competing first-line treatment options recommended in current Chinese clinical practice [12], and our results are of great significance in this health resource-limited setting. The main finding of the current analysis was that icotinib for treating newly diagnosed advanced NSCLC and harboring an EGFR mutation provides better health outcomes than other therapies. The main finding suggested that the ICER of the icotinib strategy was lower than the ICERs of the pemetrexed maintenance and gefitinib strategies. Icotinib was found to be the most cost-effective therapeutic approach. The PSA found that the highest probability of cost-effectiveness of icotinib would be achieved at a threshold of $22,200. These findings are generally coherent with previous published studies that reported the economic outcomes of TKIs (gefitinib, afatinib and erlotinib) as first-line managements for advanced NSCLC [14–16].

In the study from Japan, the marginal cost and effectiveness of the screening strategy (EGFR mutation testing followed by gefitinib treatment) per patient were estimated to be approximately $1,180 and 0.036 QALYs, respectively, leading to a marginal cost of $32,500 per additional QALY gained. These findings demonstrated that the EGFR screening strategy in Japan is cost-effective [15]. By using a decision-analytic model, one Singaporean study evaluated the economic outcome of gefitinib treatment for advanced NSCLC patients with activating EGFR mutations, which showed EGFR testing combined with first-line gefitinib treatment was a superior strategy to standard care due to lower costs and greater health benefits [17].

Our study also found that the icotinib strategy was less expensive than the pemetrexed maintenance and gefitinib strategies, which were inferior owing to their smaller health benefits and higher costs. One plausible explanation for this finding is that the cost per cycle of the icotinib strategy was lower than that of the pemetrexed maintenance and gefitinib strategies. One recently published study of second-line treatment for advanced NSCLC showed 5-year QALYs of 0.279 and 0.269 and medical costs of $10,662.82 and $13,127.57 in the icotinib and gefitinib groups, respectively, suggesting that icotinib was a less expensive strategy than gefitinib [18]. Consistent with our study, that previous study also found that the utility of PFS was a sensitive parameter.

This study has several limitations. First, owing to no clinical data evaluating these four first-line alternatives in one trial, a network meta-analysis was performed in the present study for an indirect comparison, where the patient characteristics were assumed to be similar although the moderate heterogeneity was detected. Second, a Weibull survival model was used to simulate the lifetime outcomes. This approach was another limitation of this study, although the results of the sensitivity analysis suggested that the model outcome was not sensitive to the variables related to HR of PFS. Third, some key clinical inputs, such as the survival data for the gefitinib and pemetrexed maintenance strategies, were extracted from distinct RCTs with different study designs conducted on the Western population. To minimize potential bias and uncertainty in outcomes, we tested the impact using sensitivity analyses. Fourth, the current analysis did not fully examine other potentially competing alternatives for advanced NSCLC, such as afatinib, erlotinib and bevacizumab [19–22], because these agents are not currently licensed as first-line treatments in China. Finally, the utility data was not Chinese-specific, which might lead to bias in the model outcomes. However, the sensitivity analyses showed only minor impacts of utility.

In summary, from the perspective of Chinese health care system, gene-guided icotinib therapy for newly diagnosed advanced NSCLC harboring EGFR mutations is a cost-effective alternative relative to chemotherapy containing pemetrexed and gefitinib, based on the higher efficacy and lower cost of icotinib treatment.

## MATERIALS AND METHODS

### Analytical overview and model structure

**Figure 3:**
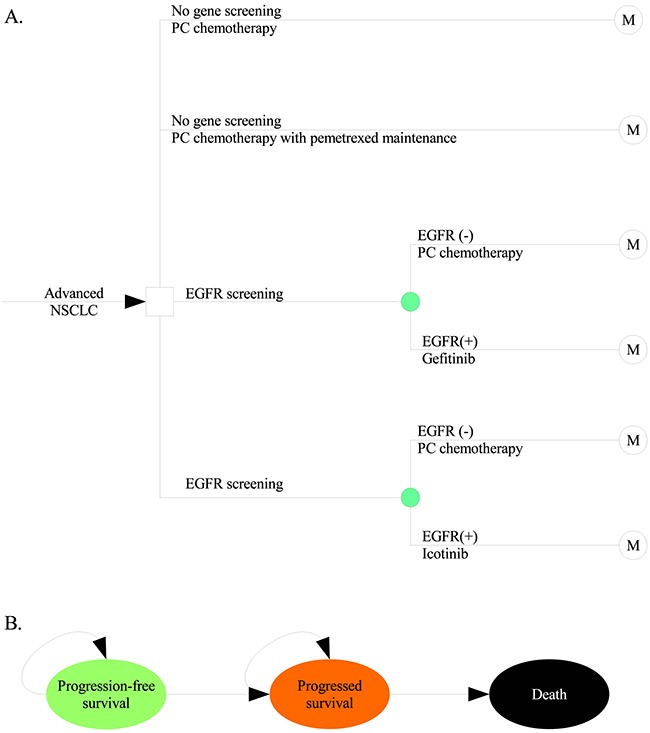
Schematics of the decision tree **A**. and the Markov state transition model **B**. NSCLC: non-small cell lung cancer; EGFR: epidermal growth factor receptor.

A mathematical model was constructed to measure the ten-year clinical and economic outcomes of first-line treatments for patients with advanced NSCLC. A hypothetical cohort with confirmed stage IIIb or IV NSCLC and positive for an EGFR mutation was created to compare four potential competing strategies: four-cycle chemotherapy based on pemetrexed plus cisplatin (PC) (control strategy), PC chemotherapy followed by maintenance with pemetrexed (pemetrexed strategy) or initial targeted treatment with gefitinib (gefitinib strategy) or icotinib (icotinib strategy) (Figure 3A). After the disease progressed, salvage chemotherapy or supportive care was administered. Health and economic outcomes were projected using a Markov process (Figure 3B) considering three exclusive health states: PFS, survival after progression and death. The Markov cycle length was 21 days, and the initial health state for all patients was PFS. The risk of disease progression or death was determined based on the published literature. Owing to the good tolerability of pemetrexed, gefitinib and icotinib [23–25], this hypothetical analysis did not consider the impact of tolerability to simplify the model.

The primary outcomes were disease-free life years (LYs), overall LYs, quality-adjusted life-years (QALYs) and cost. Cost and QALYs were discounted at an annual rate of 5%, in line with Chinese guidelines for pharmacoeconomic evaluations. The costs are shown in 2015 US dollars. Incremental cost-effectiveness ratios (ICERs), presented as cost per additional QALY gained, were examined. We used 3× the per capita gross domestic product (GDP) of China in 2015 (US $22,200) as the cost-effectiveness threshold according to the WHO recommendations.

### Clinical data

The transition parameters and proportions were based on meta-analyses or RCTs to the greatest possible extent. The PARAMOUNT trial reported the efficacy of pemetrexed maintenance therapy after PC-based induction chemotherapy in patients with non-squamous NSCLC. The results showed a median PFS time of 6.9 months for the pemetrexed group and 5.6 months for the placebo group (HR 0.59, 95% CI 0.47–0.74); in that study, PFS was measured from the beginning of induction treatment (rather than from the time of randomization) [24]. The CONVINCE trial compared first-line icotinib therapy to PC followed by maintenance therapy with pemetrexed in lung adenocarcinoma patients harboring a sensitizing EGFR mutation; the results of that trial showed that the icotinib therapy significantly prolonged PFS compared with the chemotherapy (296 days vs 219 days; HR 0.67, 95% CI 0.49-0.90, p = 0.008) [23]. The IPASS trial demonstrated the superiority of gefitinib to traditional (pemetrexed-free) chemotherapy in terms of PFS in patients harboring EGFR mutations (HR 0.48, 95% CI 0.36-0.64). The subsequent NEJ002 and WJTOG3405 trials, which included only patients harboring EGFR mutations, further confirmed that gefitinib is superior to (pemetrexed-free) chemotherapy in terms of PFS (HR 0.30, 95% CI 0.22-0.41 in the NEJ002 trial, and HR 0.49, 95% CI 0.33-0.71 in the WJTOG3405 trial) in patients harboring EGFR mutations [25–27]. One meta-analysis reported that the HR of PFS for pemetrexed plus platinum doublet chemotherapy relative to platinum plus another first-line chemotherapy agent as a first-line treatment for advanced non-squamous NSCLC patients was 0.90 (95% CI 0.80–1.01) [28].

**Figure 4:**
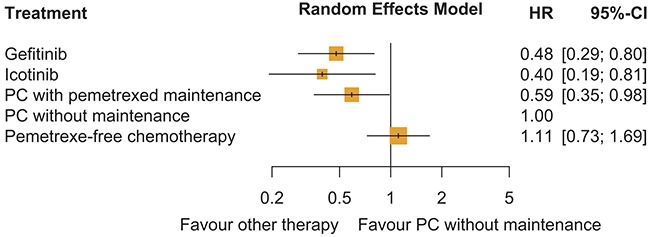
Random-effects network meta-analysis results for PFS of interest using a binomial logit model PC: pemetrexed plus cisplatin.

Indirect comparisons of the four strategies were conducted using the survival rate for PC chemotherapy (control strategy) from the reports of the PARAMOUNT trial as the reference [24]. Weibull survival models were fitted to the Kaplan-Meier curves of PFS for the control strategy according to data from the reports of the PARAMOUNT trial. The estimated Weibull scale (λ) and shape (γ) parameters are shown in Table 2. The survival probability at time *t* was calculated using the following formula: *S*(*t*) = P(*T≥t*) = exp(*-λt^γ^*). The transition probability at a given cycle *t* was calculated using the following formula: P(*t*) = 1 - exp[*λ(t - 1)^γ^ - λt^γ^*]. The Weibull survival curves of the three alternative strategies were derived using the adjusted Weibull scale (*λ_alternative strategy_* = *λ_control strategy_*×*HR_network meta-analysis_*) and shape (*γ_alternative strategy_* = *γ_control strategy_*) parameters, as previously described in published studies [29, 30]. However, given the absence of head-to-head clinical trial data, the HRs of PFS for the gefitinib and icotinib strategies relative to the control strategy considered in the economic model were generated using network meta-analysis based on a random-effects model because of the heterogeneity (*I*^2^ = 64.1%). As showed in Figure 4, this network meta-analyses was performed with a graph-theoretical methodology implemented in the R package netmeta [31, 32]. The HRs of PFS from each clinical trial were derived from the previously mentioned published studies. Table S1 in Appendix summarized the characteristics of all involved studies.

**Table 2:** Key clinical data

Parameter	Expected Values (Ranges)	Distribution (Parameters)	Description and Reference
Weibull survival model of PFS for PC	Scale = 0.1029; Shape = 1.3077; r^2^ = 0.981	NA	[24]
HR of PFS for PC followed by pemetrexed maintenance	0.59	Normal (0.59, 0.161)	Network meta-analysis
HR of PFS for gefitinib	0.48(0.29-0.8)	Normal (0.48, 0.13)	Network meta-analysis
HR of PFS for icotinib	0.4(0.19-0.81)	Normal (0.4, 0.158)	Network meta-analysis
Probability of survival after progression	0.086(0.08-0.093)	Beta (751.1, 7982.7)	[33]
EGFR mutation prevalence	0.47(0.2-0.76)	Normal (0.47, 0.143)	[34]
Probability of SAEs from the control strategy	0.456(0.342-0.57)	Beta (33.6, 40.1)	[24, 35]
Probability of SAEs from the pemetrexed strategy	0.637(0.478-0.796)	Beta (22.4, 12.8)	[24]
Probability of SAEs from the gefitinib strategy	0.1(0.075-0.125)	Beta (53.3, 479.3)	[36]
Probability of SAEs from the icotinib strategy	0.07(0.053-0.088)	Beta (56.3, 747.4)	[36]
Body surface area (m^2^)	1.72(1.5-1.9)	Normal (1.72, 0.102)	[37]

PC: pemetrexed plus cisplatin; EGFR: epidermal growth factor receptor; SAEs: serious adverse events (≥ grade 3); PFS: progression-free survival; HR: hazard ratio; NA: not applicable.

The probability of survival after progression was derived from a published study [33], which identified 69 trials of first-line chemotherapy for advanced NSCLC and which found that the median survival time after disease progression was 5.4 months. The EGFR mutation frequency among NSCLC patients in the Asia-Pacific region was 47% (20%-76%) [34].

### Cost and utility data

This analysis considered the setting of the Chinese health care system. Only direct medical costs, including the costs of EGFR mutation testing, first and second-line chemotherapies (including prescription, preparation, and administration), concomitant medications during therapy, management of treatment-related SAEs, and routine follow-up and laboratory testing (Table 3), were included in the model.

**Table 3:** Base-Case Costs Estimates and Utilities

Parameter	Expected Values (Ranges)	Distribution (Parameters)	Description and Reference
Costs (US $)			
Cost of pemetrexed per 500 mg	967.57 (533.02-2126.51)	Gamma (2303, 0.42)	[43]
Cost of the chemotherapy excluding the non-platinum agents per 21-day cycle	518.4 (388.8-648)	Gamma (4064.3, 0.13)	[43]
Cost of icotinib per day	31.72 (15.86-31.72)	Gamma (248.7, 0.13)	Local charge
Cost of gefitinib per day	37.43 (18.71-37.43)	Gamma (293.4, 0.13)	Local charge
Cost of follow-up per unit	55.6 (41.7-69.4)	Gamma (437.5, 0.13)	[43]
Cost of salvage chemotherapy per cycle	2352.7 (1921.1-4383.3)	Gamma (8812.4, 0.27)	[43]
Cost of palliative care in end-of-life	2042.91 (793.65-5456.19)	Gamma (3508.8, 0.58)	[43]
Cost of supportive care per cycle	337.5 (158.7-793.7)	Gamma (703.2, 0.48)	[43]
Cost of SAEs per unit	507.4 (189.7-825.0))	Gamma (1588.6, 0.32)	[43]
Cost of EGFR mutation testing	380.95 (158.73-476.19)	Gamma (1792, 0.21)	Local charge
Utilities			
Utility of PFS	0.82 (0.78-0.86)	Beta (373.6, 82)	[44, 45]
Utility of OS	0.58 (0.5-0.66)	Beta (84, 60.9)	[44, 45]
Disutility of SAEs	0.35 (0.31-0.39)	Beta (199.1, 369.7)	[44]

EGFR: epidermal growth factor receptor; SAEs: serious adverse events (≥grade 3); PFS: progression-free survival; OS: overall survival.

Icotinib at a dose of 375 mg per day or gefitinib at a dose of 250 mg per day was assumed to be administered to patients positive for an EGFR mutation until disease progression [23, 25–27]. Chemotherapy (pemetrexed, 500 mg/m^2^ of body surface area (BSA), plus cisplatin, 75 mg/m^2^) was administered every 21 days for four cycles. Because generic pemetrexed was widely used in Chinese clinical practice, we used the cost of generic pemetrexed in the base-case analysis. In the pemetrexed strategy, pemetrexed treatment (500 mg/m^2^ every 21 days) was continued in patients who did not progress after four cycles of induction [24]. After disease progression, salvage chemotherapy and supportive care were prescribed; in this model, 56.6% (26%-72%) of patients received salvage treatment regardless of the first-line therapy [24, 38–42]. The costs of utilizing resources related to salvage chemotherapies, management of SAEs, supportive care and palliative care in end-of-life were derived from a previously published study [43]. The costs for management of SAEs from each strategy were calculated as the cumulative probability-weighted average of SAE costs from the first-line control strategy using the following formula: cost of SAEs from the platinum-based chemotherapy per cycle × cumulative probability of SAEs from the corresponding strategy / cumulative probability of SAEs from the platinum-based chemotherapy [43]. To calculate the dosage of chemotherapeutic agents, we assumed that a typical patient had a weight of 65 kg and a height of 1.64 m, resulting in a BSA of 1.72 m^2^. The cost of EGFR mutation testing per patient was provided by the laboratories of local hospitals. The treatment costs were estimated based on a clinical study.

Because of the high costs of icotinib and gefitinib, as well as the limited wealth of patients in China, the icotinib and gefitinib Patient Assistance Program (PAP) was implemented for Chinese patients positive for ALK gene rearrangement. Currently, the PAP requires patients to pay US $11,538 for gefitinib and US $11,077 for icotinib, after which they receive icotinib and gefitinib for free until disease progression. Therefore, the impact of the PAP was evaluated in scenario analyses.

The utility scores of PFS and survival after progression were obtained from previously published studies (Table 2) [44, 45]. The reported disutility caused by SAEs was also considered in the current analysis [44].

### Sensitivity analyses

One-way and probabilistic sensitivity analyses were performed to examine the uncertainty in the model. In the one-way sensitivity analyses, relevant parameters were changed one-by-one to their respective upper and lower boundaries in order to explore the sensitivity of the findings to plausible variations in specific data inputs. The results of the one-way sensitivity analyses are presented in a Tornado diagram. The ranges of the parameters used in the one-way sensitivity analyses were obtained from the published literature; when reported data were not available, a range of ±25% of the base-case value was used (Table 1 and 2). For the probabilistic sensitivity analyses, parameters were sampled using the Monte Carlo method to run 1,000 replicated outcomes. Based on the ISPOR-SMDM Modeling Good Research Practices Task Force report on model parameter estimation and uncertainty [46], the beta distribution was used for incidence rates, risks, probabilities, proportions and utilities; the normal distribution was used for the HR; and the gamma distribution was used for costs (Table 1 and 2). Cost-effectiveness acceptability curves (CEACs) of vaccination versus no vaccination were generated to present the probabilities of cost-effectiveness. Model development and data analysis were performed in the R statistical environment (version 3.3.1; R Development Core Team, Vienna, Austria).

## SUPPLEMENTARY APPENDIX TABLE




