# Supplementary material for: Cost-effectiveness of gefitinib, icotinib, and pemetrexed-based chemotherapy as first-line treatments for advanced non-small cell lung cancer in China
Source: Oncotarget. 2016 Dec 27;8(6):9996–10006. doi: 10.18632/oncotarget.14310 (PMC5354787; doi:10.18632/oncotarget.14310)
Supplement: Supplementary file 1 [file oncotarget-08-9996-s001.pdf]

Cost-effectiveness of gefitinib, icotinib, and pemetrexed-based chemotherapy as first-line treatments for advanced non-small cell lung cancer in China

APPENDIX TABLE

Appendix Table 1: Characteristics of included studies.

See Supplementary File 1
