# Supplementary material for: Cost-effectiveness of gefitinib, icotinib, and pemetrexed-based chemotherapy as first-line treatments for advanced non-small cell lung cancer in China
Source: Oncotarget. 2016 Dec 27;8(6):9996–10006. doi: 10.18632/oncotarget.14310 (PMC5354787; doi:10.18632/oncotarget.14310)
Supplement: Supplementary file 2 [file oncotarget-08-9996-s002.docx]

| Table 1: Characteristics of included studies | | | | | | | |
| --- | --- | --- | --- | --- | --- | --- | --- |
| Studies | Active treatment arm | Control arm | Year | Sample size | Patients status | PFS(Months) | HR of PFS |
| NEJ002[1] | Gefitinib | TC | 2010 | 228 | Chemotherapy-naive | 10.8 vs. 5.4 | 0.32 |
| WJTOG 3405[2] | Gefitinib | DP | 2010 | 172 | Chemotherapy-naive | 9.6 vs. 6.6 | 0.52 |
| IPASS[3] | Gefitinib | TC | 2009 | 1217 | Chemotherapy-naive | 9.8 vs. 6.4 | 0.48 |
| CONVINCE[4] | Icotinib | PC induction+ Pemetrexed maintenance treatment | 2016 | 296 | Chemotherapy-naive | 9.9 vs. 7.3 | 0.67 |
| PARAMOUNT [5] | PC induction+ Pemetrexed maintenance treatment | PC induction treatment | 2012 | 539 | Chemotherapy-naive | 9.9 vs. 7.4 | 0.62 |
| Scagliotti[6] | PC induction treatment | GC | 2008 | 1725 | Chemotherapy-naive | 4.8 vs. 5.1 | 1.08 |
| PC: pemetrexed+cisplatin; TC, carboplatin plus palitaxel; GP, cisplatin plus gemcitabine; DP, cisplatin plus docetaxel. | | | | | | | |

Reference:

1 Maemondo M, Inoue A, Kobayashi K, Sugawara S, Oizumi S, Isobe H, Gemma A, Harada M, Yoshizawa H, Kinoshita I, Fujita Y, Okinaga S, Hirano H, Yoshimori K, Harada T and Ogura T, et al. Gefitinib or chemotherapy for non-small-cell lung cancer with mutated EGFR. N Engl J Med. 2010; 362(25):2380-2388.

2 Mitsudomi T, Morita S, Yatabe Y, Negoro S, Okamoto I, Tsurutani J, Seto T, Satouchi M, Tada H, Hirashima T, Asami K, Katakami N, Takada M, Yoshioka H, Shibata K and Kudoh S, et al. Gefitinib versus cisplatin plus docetaxel in patients with non-small-cell lung cancer harbouring mutations of the epidermal growth factor receptor (WJTOG3405): an open label, randomised phase 3 trial. LANCET ONCOL. 2010; 11(2):121-128.

3 Mok TS, Wu YL, Thongprasert S, Yang CH, Chu DT, Saijo N, Sunpaweravong P, Han B, Margono B, Ichinose Y, Nishiwaki Y, Ohe Y, Yang JJ, Chewaskulyong B, Jiang H and Duffield EL, et al. Gefitinib or carboplatin-paclitaxel in pulmonary adenocarcinoma. N Engl J Med. 2009; 361(10):947-957.

4 Shi Y, Wang L, Han B, Li W, Yu P, Liu Y, Ding C, Song X, Ma Z, Ren X, Feng J, Zhang H, Chen G, Wu N, Han X and Yao C, et al. First-Line Icotinib Versus Cisplatine/Pemetrexed Plus Pemetrexed Maintenance in Advanced NSCLC Patients with EGFR Mutation. J THORAC ONCOL. 2015; 102(9):S205-S206.

5 Paz-Ares L, de Marinis F, Dediu M, Thomas M, Pujol JL, Bidoli P, Molinier O, Sahoo TP, Laack E, Reck M, Corral J, Melemed S, John W, Chouaki N, Zimmermann AH and Visseren-Grul C, et al. Maintenance therapy with pemetrexed plus best supportive care versus placebo plus best supportive care after induction therapy with pemetrexed plus cisplatin for advanced non-squamous non-small-cell lung cancer (PARAMOUNT): a double-blind, phase 3, randomised controlled trial. LANCET ONCOL. 2012; 13(3):247-255.

6 Scagliotti GV, Parikh P, von Pawel J, Biesma B, Vansteenkiste J, Manegold C, Serwatowski P, Gatzemeier U, Digumarti R, Zukin M et al: Phase III study comparing cisplatin plus gemcitabine with cisplatin plus pemetrexed in chemotherapy-naive patients with advanced-stage non-small-cell lung cancer. J CLIN ONCOL 2008, 26(21):3543-3551.
